# Transcriptional Profiling of Rats Subjected to Gestational Undernourishment: Implications for the Developmental Variations in Metabolic Traits

**DOI:** 10.1371/journal.pone.0007271

**Published:** 2009-09-29

**Authors:** Tiffany J. Morris, Mark Vickers, Peter Gluckman, Stewart Gilmour, Nabeel Affara

**Affiliations:** 1 Department of Pathology, University of Cambridge, Cambridge, England; 2 Liggins Institute and National Research Centre for Growth and Development, University of Auckland, Auckland, New Zealand; Sapienza University of Rome, Italy

## Abstract

A link has been established between prenatal nutrition and the development of metabolic and cardiovascular diseases later in life, a process referred to as *developmental programming*. It has been suggested that the trajectory of development is shifted by alterations in the maternal nutritional state leading to changes in developmental plasticity, in part underpinned by epigenetic changes in gene regulation. However, to date, only candidate gene approaches have been used to assess expression and molecular changes in the offspring of maternally undernourished animals. Furthermore, most work has focused on animals at an age where the programmed phenotype is already manifest and little is known about changes in gene expression in the offspring *prior* to development of obesity and related metabolic disorders. Gene expression profiles of liver, retroperitoneal white adipose fat, and biceps femoris skeletal muscle tissue from young adult male rats (55 days old) in which nutritional status had been manipulated in utero by maternal undernutrition (UN) were compared to the profiles of offspring of *ad libitum* fed mothers serving as the control group (AD) (8 offspring/group). The expression profiles were determined using the Illumina RatRef-12 BeadChip. No significant changes in expression were identified for skeletal muscle or white adipose tissue. However, studies of liver tissue showed 249 differentially expressed genes (143 up regulated, 106 down regulated). Although the animals at day 55 have yet to develop obesity they already show biochemical abnormalities and by day 110 express a phenotype characterized by increased adiposity and altered insulin sensitivity. An analysis of pathways affected suggests that intrauterine programming of UN animals to favor fat as an energy source results in mitochondrial dysfunction which initially affects the postnatal hepatic function and subsequently, via the resultant metabolic changes in other organs leads to the evolution of a phenotype similar to that of the metabolic syndrome.

## Introduction

Developmental plasticity is the process by which environmental factors acting during critical windows in early life affect the developmental trajectory and thus the mature phenotype. Such changes are often irreversible and are generally considered to be underpinned at least in part by epigenetic processes [1], [2]. While adaptive in origin developmental plasticity can have maladaptive consequences [3] and thus in humans can affect health [4].

The developing fetus is completely dependent on its mother and so it follows that maternal nutrition during pregnancy, in particular, has a strong influence on the intrauterine environment. The DOHaD (Developmental Origins of Health and Disease) hypothesis proposes that the metabolic syndrome (i.e. coronary heart disease, type 2 diabetes, stroke, hypertension) originates through developmental plasticity in response to undernutrition during fetal life and infancy [1], [5], [6]. It has been hypothesized that these diseases of later life arise from a mismatch [7] between the actual postnatal environment and that predicted by the fetus during the phase of developmental plasticity [8], [9]. A variety of maternal nutritional manipulations in the rat have induced offspring who develop obesity, insulin resistance, and endothelial dysfunction in later life including global maternal undernutrition as used in this study [10] and maternal low protein by isocaloric diets [11].

The mechanistic basis of such changes is only now being explored. It has been proposed that epigenetic modifications of the fetal genome establish new patterns of gene expression that reset the metabolic state that is maintained into adulthood [12]. Effects on candidate genes using such rat models have focused on genes known to be significant in the metabolic syndrome including peroxisomal proliferator-activated receptor (PPARα) and the glucocorticoid receptor (GR) [11], [13], [14]. These observations suggest that there are methylation differences in the promoters of these genes and changes in mRNA levels between the offspring of normally and undernourished mothers. A broader picture of the cellular functions and genetic pathways that may be implicated can be obtained using a non-biased, global microarray approach to study gene expression in key tissues between offspring of control and undernourished mothers.

In the present study, an established rat model of balanced maternal undernutrition [10] has been used for investigation of gene expression differences in target tissues (liver, retroperitoneal white adipose fat, and biceps femoris skeletal muscle) between offspring of control and undernourished mothers. We studied young adult rats at day 55 of age when they have not yet developed the abnormal phenotype in order to identify any gene expression changes that may play a role in predisposing these animals to the development of the metabolic syndrome. The findings show that no significant gene expression changes are detected between the two groups in skeletal muscle and white adipose tissue. However, the liver reveals 249 significant gene expression differences (p<0.05) showing significant down regulation of expression of several key genes affecting the entry of glucose into the cell and its metabolism via the glycolytic and tricarboxylic acid pathways. This would result in a substantial impairment in their ability to utilize carbohydrate on restoration of a normal diet. In addition, there was a marked increase in expression of genes associated with the intracellular trafficking of fatty acids, their peroxisomal degradation and their entry into the mitochondrion to undergo β-oxidation. This might suggest that following intrauterine undernutrition these animals have lost the flexibility to switch between carbohydrate and fat as energy sources. The analysis also reveals a substantial impairment of the electron transport chain as evidenced by a down regulation in expression of 3 components of complex I and the ATP synthase that generates ATP from the proton gradient. There is also a deficit in expression of a key enzyme in the biosynthesis of the NAD proton carrier.

We suggest that maternal undernutrition leads to offspring that favor fat as an energy source thus resulting in mitochondrial dysfunction that initially affects postnatal hepatic function and subsequently, other organs.

## Materials and Methods

### Animals

This study exploited a well defined model of developmental programming via maternal undernutrition [10], [15]. Briefly, virgin Wistar rats (age, 100±5 days) were time mated using a rat estrous cycle monitor to assess the stage of estrous of the animals before introducing the male. After confirmation of mating, rats were housed individually in standard rat cages with free access to water. All rats were kept in the same room with constant temperature maintained at 25°C and a 12-h light/12-h darkness cycle. Animals were assigned to one of two nutritional groups: a) undernutrition (30% of *ad libitum*) of a standard chow diet throughout gestation (UN group), b) standard chow diet *ad libitum* throughout gestation (AD group). Food intake and maternal weights were recorded daily until the end of pregnancy. After birth, pups were weighed and litter size was adjusted to 8 pups per litter to assure adequate and standardized nutrition until weaning. Pups from undernourished mothers were cross-fostered onto dams that had received AD feeding throughout pregnancy. At weaning (day 22), animals were weight matched within maternal dietary group to 2 animals per cage. Eight animals per group were culled at day 55 following an overnight fast to represent an age known to precede the development of increased adiposity and altered insulin sensitivity in UN offspring. Tissues (liver, retroperitoneal white adipose fat, and biceps femoris skeletal muscle) were immediately dissected and snap-frozen in liquid nitrogen for molecular analysis. A subcohort of animals (n = 8 per group) were maintained until postnatal day 110 to represent an age where phenotypic changes in growth and metabolism between AD and UN offspring have previously been reported. [10], [16] The Animal Ethics Committee of The University of Auckland approved all animal manipulations.

### Phenotypic Measurements

Serial dual-energy x-ray absorptiometry (DEXA) analysis was performed on the animals at day 55 and day 110 to establish body composition and assessment of bone parameters. In addition to DEXA, body adiposity was assessed via standard techniques (fat depot dissection and weighing) following post-mortem. Body weight and food and water intake were monitored. Plasma analysis incorporated IGF-1, insulin, C-peptide, and lipid profiles. Statistical analyses were performed with the Knowledge Discovery Environment (InforSense Ltd., London, UK) statistical package. Differences between groups were determined by a T-test for difference in variance with an FDR correction, and data are shown as an adjusted p-value±standard error.

### Microarray Hybridization and Analysis

Global gene expression analysis was performed using the Illumina Bead Array Platform. Briefly, total RNA was isolated from cells with TRIzol reagent (Invitrogen, Paisley, Scotland, UK) followed by column purification (RNeasy, Qiagen, UK). RNA quality was assessed with the Lab-on-a-chip system (Agilent, Palo Alto, CA). The RNA samples were amplified following the Illumina TotalPrep RNA amplification protocol. The fluorescent labeling and hybridization of RNA samples were also performed according to custom protocols defined in the manufacturer's instructions. The samples from liver, skeletal muscle, and white adipose fat tissue for male offspring (n = 8) were hybridized to the RatRef-12 BeadChip available from Illumina (sampling 21,290 genes from the rat transcriptome). The hybridization signals were captured and quantified using a Bead Array scanner and the associated Bead Studio software. Bead Studio includes a spectrum of internal controls for determining data quality. These check for consistency in signal intensity, expected background and noise levels, mismatches versus perfect matches, and signal in housekeeping genes (Figure S1A-S1H). Data was exported and normalization was performed in R (Bioconductor) using the Limma [17] and Lumi [18] Bioconductor packages for microarray analysis. Data was filtered for those showing expression on at least one array and a variance-stabilizing transformation [19] was done to take advantage of the multiple technical replicates (on average 20–30 per array) for each gene available on each Illumina chip. Quantile normalization was chosen to remove technical variability between arrays. Density plots of intensity and box plots of amplitude were used to visualize the raw data compared to the filtered and normalized data (Figure S2A-S2D). Limma was used for pairwise comparisons between AD and UN using a linear model to compute p-values, which were adjusted using the Benjamini and Hochberg multiple testing correction. The derivation of the p-value reflects the degree of variance between biological replicates and thus is a measure of the confidence in assigning significance of small fold changes in gene expression. Genes with a p-value<0.05 were considered to be significantly differentially expressed. Gene ontology analysis was done using DAVID: Database for Analysis, Visualization, Integrated Discovery [20] and Ingenuity Pathway Analysis software (Ingenuity, Stanford USA).

### Quantitative Real-Time PCR

**Figure 1:**
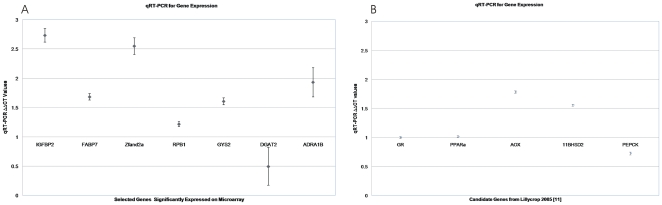
qRT-PCR for Microarray Verification. QRT-PCR for the two treatments was run in triplicate with cyclophilin as a control. The ΔΔCT values are shown for each gene along with error bars for standard error. (A) Seven genes were chosen from the 249 significantly differentially expressed genes on the Illumina microarray. The RNA from the 8 biological replicates was pooled for each of the two treatment groups. In Table 2, the ΔΔCT values have been converted into a fold change for comparison to the array results. (B) Five Previously Published Candidate Genes from Lillycrop et al. [11] were analyzed be qRT-PCR. Each of the eight individual samples was run in triplicate (24 data points for each treatment group). Due to the large number of data points, the error bars are very small and difficult to see.

The comparative expression levels in AD and UN RNA of seven genes identified as differentially expressed from our microarray data were verified by qRT-PCR, together with the expression levels of five genes not present on the array. Cyclophilin was used for the generation of a standard curve and normalization of the RNA concentration. Each qRT-PCR was performed in triplicate. Real-time RT-PCR was performed in 96-well white plates (Abgene) using the Verso SYBR Green 2-Step qRT-PCR Fluorescein Kit (Thermo Scientific, UK) according to the manufacturer's protocols; the resulting fluorescence was quantified using an iCycler system (Bio-Rad). The threshold cycle value (C_T_) was obtained for each well as the cycle number at which the measured fluorescence crossed the arbitrary threshold of 150 units (all values are in log phase). The average C_T_ was calculated for each gene in each sample. Data were normalized to cyclophilin, with ΔC_T_ calculated as follows: ΔC_T_  =  C_T_(test)−C_T_(cyclophilin). ΔΔC_T_ values were then calculated as the change in ΔC_T_ for the UN sample relative to the ΔC_T_ value for the AD sample using the equation: ΔΔC_T_  = 2^CT(test)^−2^CT(cyclophilin)^. This data is plotted in Figure 1 and primer sequences are shown in Table S1.

## Results

### Phenotypic Assessment

For each of the 8 animals in each of the AD and UN groups, a series of phenotypic measurements relevant to the metabolic syndrome was recorded at day 55 (Table 1), the age at which gene expression analysis was performed, and in a subcohort of animals at day 110 (Table 2). From these measurements, it is clear that at day 55 there are very few differences in key indicators (insulin resistance, glucose intolerance, elevated blood lipids and increased adiposity) as might be expected at this early stage in the progression of the metabolic syndrome. However, by day 110 there are clear indications of abnormalities in insulin sensitivity and markers of adiposity including total fat mass and plasma leptin concentrations.

**Table 1:** Phenotype data, day 55 offspring.

	AD mean	UN mean	Adjusted p-value
Body weight (grams)	282.38	234.50	0.001
Body length (mm)	203.63	192.63	0.005
Liver weight (% body weight)	3.65	3.02	0.014
Spleen weight (% body weight)	0.32	0.27	NS
Heart weight (% body weight)	0.36	0.71	NS
Fat pad weight (% of body weight)	0.80	0.59	0.039
Plasma glucose (mmol/l)	6.24	6.67	NS
Urea (mmol/l)	4.76	6.18	0.010
Free Fatty Acids (mmol/l)	0.79	0.68	NS
Glycerol (mmol/l)	0.21	0.19	NS
Total Protein (g/DL)	5.45	5.39	NS
Lipase (U/l)	9.10	9.06	NS
C-Peptide (pg/ml)	132.18	162.85	NS
Triglycerides (mmol/l)	0.78	0.77	NS
IGF-1 (ng/ml)	1221.50	1277.63	NS
Creatinine (mmol/l)	20.15	23.64	NS
Insulin (ng/ml)	0.33	0.53	NS
Total Fat (%)	24.09	23.66	NS
LDL (mmol/l)	0.27	0.46	0.006
HDL (mmol/l)	1.27	1.34	NS
LDL∶HDL ratio	0.21	0.35	0.003

Phenotypic measurements relevant to metabolic syndrome measured for each of the eight animals in each treatment group at day 55. The p-value was calculated with a t-test and an FDR correction. NS  =  not significant.

**Table 2:** Phenotype data, day 110 male offspring.

	AD mean	UN mean	Adjusted p-value
Body weight (grams)	511	489	NS
Body length (mm)	238	218	0.05
Total Fat (%)	27.8	35.6	0.05
Fat pad weight (% of body weight)	1.45	2.14	0.05
Leptin (ng/ml)	9.6	22.5	0.005
Insulin (ng/ml)	0.260	0.502	0.001
C-Peptide (pg/ml)	466	729	0.05
Plasma glucose (mmol/l)	7.0	7.2	NS
Free Fatty Acids (mmol/l)	0.86	0.99	NS
Liver weight (% body weight)	2.92	2.78	NS
IGF-1 (ng/ml)	1236	1145	NS

Phenotypic measurements relevant to metabolic syndrome measured for each of the eight animals in each treatment group at day 110. The p-value was calculated with a t-test and an FDR correction. NS  =  not significant.

### Expression Analysis

Expression profiling was done for liver, white adipose fat, and skeletal muscle tissue from young adult (55-day-old) male rats born to either AD or UN dams. In each group there were eight biological replicates, all of which were raised postnatally on an *ad libitum* diet. Expression profiles were determined using the Illumina RatRef-12 BeadChip that permits interrogation of 21,290 rat genes. Genes with a p-value<0.05 were considered to show significant differential expression between the two groups. Using these criteria, no significant gene expression changes between the two groups were identified for skeletal muscle or white adipose tissue in the day 55 animals. However, the comparison for liver revealed a list of 249 differentially expressed genes. Table S2 summarizes the expression values for all genes represented on the array and Table S3 the list of 249 differentially expressed genes with associated p-values and fold changes between the two groups.

#### Quantitative RT-PCR (qRT-PCR) Analysis of Selected Genes

In order to confirm the expression data derived from array analysis, seven genes (*IGFBP2* (NM_013122), *FABP7* (NM_030832), *Zfand2a* (NM_001008363), *DGAT2* (XM_574498), *GYS2* (NM_013089), *ADRA1B* (NM_016991), *and RBP1* (XM_230637)) found to be differentially expressed were selected for qRT-PCR on the pooled RNA samples for the biological replicates in each treatment group. Cyclophilin was used as an invariant control for normalization. Figure 1A summarizes the data and illustrates that the differences in ΔΔCt values between the AD and UN RNA populations for each gene are consistent with the expression changes determined by array analysis. These values were then converted to fold change to directly compare the qRT-PCR values to the expression values from the microarray (Table 3).

**Table 3:** Fold Change Values Comparing Microarray and QRT-PCR.

Gene Symbol	Genbank Accession Number	Microarray Expression (Fold Change)	QRT-PCR Expression (Fold Change)
ADRA1B	NM_016991.2	0.735	1.932
DGAT2	XM_574498.1	0.764	0.494
FABP7	NM_030832.1	1.381	1.682
GYS2	NM_013089.1	1.541	1.606
IGFBP2	NM_013122.1	2.144	2.732
Zfand2a	NM_001008363.1	1.492	2.549
RPB1	XM_230637.3	1.631	1.217

Fold change for the QRT-PCR data and the Illumina expression data is compared for each of the seven genes from the Illumina arrays that were analyzed by QRT-PCR. Fold changes for the RT-PCR reflect directly the ΔΔCt values between the two groups after normalization.

Previous analysis has provided evidence that the expression of the PPARα, GR and AOX (acyl-CoA oxidase) genes is altered in the liver of offspring from normally nourished mothers compared to mothers who had either a low protein diet or balanced undernutrition during pregnancy [11], [21]. It has also been suggested that PEPCK (phosphoenolpyruvate carboxykinase) and 11β-HSD2 (11β-hydroxysteroid dehydrogenase type 2) may also be genes influenced by diet [22], [23]. As these genes were not represented by any of the oligonucleotides on the RatRef-12 array, they were assessed by qRT-PCR in the AD and UN RNA populations using cyclophilin as the normalization control. The findings are shown in figure 1B for the 8 biological replicates in each treatment group. For PPARα and PEPCK, there is no evidence for any differences in expression levels between the two treatment groups. However, GR, AOX and 11β-HSD2 show between 1.5 to 2-fold increases in expression in the UN treatment group.

## Discussion

Animals at postnatal day 55 were chosen for gene expression analysis because they had completed puberty but had not begun to exhibit the features of the metabolic syndrome phenotype as observed later in adulthood and, as shown in the present study, as early as day 110 postnatally [10], [24]. Our results presented show that while there are differences in gene expression in hepatic tissue in day 55 males, there are no observed differences in white adipose fat or skeletal muscle. This is an intriguing finding, as both fat and skeletal muscle is adversely affected in the adult. This suggests that there is a cascade of biological processes that amplifies over time to lead to the phenotypic abnormalities. Previously Ozanne *et al.* [25] has shown, in a different but related experimental model of maternal low protein intake, that the expression of a limited number of genes in skeletal muscle is minimal early in life and accumulates later in life. It would appear that major changes in gene expression in the liver precede those in other metabolically relevant tissues.

The data for all three tissues is derived from 8 biological replicates in each treatment group with technical replication on each array varying from 20–30 fold. This means that for each gene represented on the array, there are between 160 and 240 measurements of expression level. This permits an accurate estimate of the variance that is used to derive the final p-value and provides confidence in the detection of small gene expression changes. Thus the changes found in liver are based on many measurements and are highly significant. This is further underscored by the absence of any significant changes in gene expression between the two groups in skeletal muscle or white adipose fat tissue subjected to the same analysis. The qRT-PCR analysis of candidate genes (PPARα and PEPCK) showed very little difference in expression levels between the two groups in the day 55 male offspring. This contrasts with the 10-fold, 3-fold and 3-fold increase for PPARα, GR and AOX, respectively, in the day 34 offspring born to nutritionally restricted mothers as reported by Lillycrop *et al.* [11]. Four factors may explain this difference. First, the restricted diet is different (50% protein restriction only) whereas the diet in the study reported here is 30% of normal chow for all nutrients and may impact differently on gene expression. Second, in the Lillycrop *et al*. study the animals were weaned on day 28 and spent only 6 days in the post-weaning period compared to 33 days in this present study. Third, the composition of the post-weaning diet in the present study is grain-based as opposed to a purified diet containing 50% sucrose in the Lillycrop *et al*. study. Finally, the male and female tissues were pooled in the Lillycrop *et al*. study and there may be gender differences we have not explored. Similar to previous reports, GR, 11β-HSD2 and AOX show increases of between 1.5 to 2-fold in the offspring of undernourished mothers used in this study [11].

**Figure 2:**
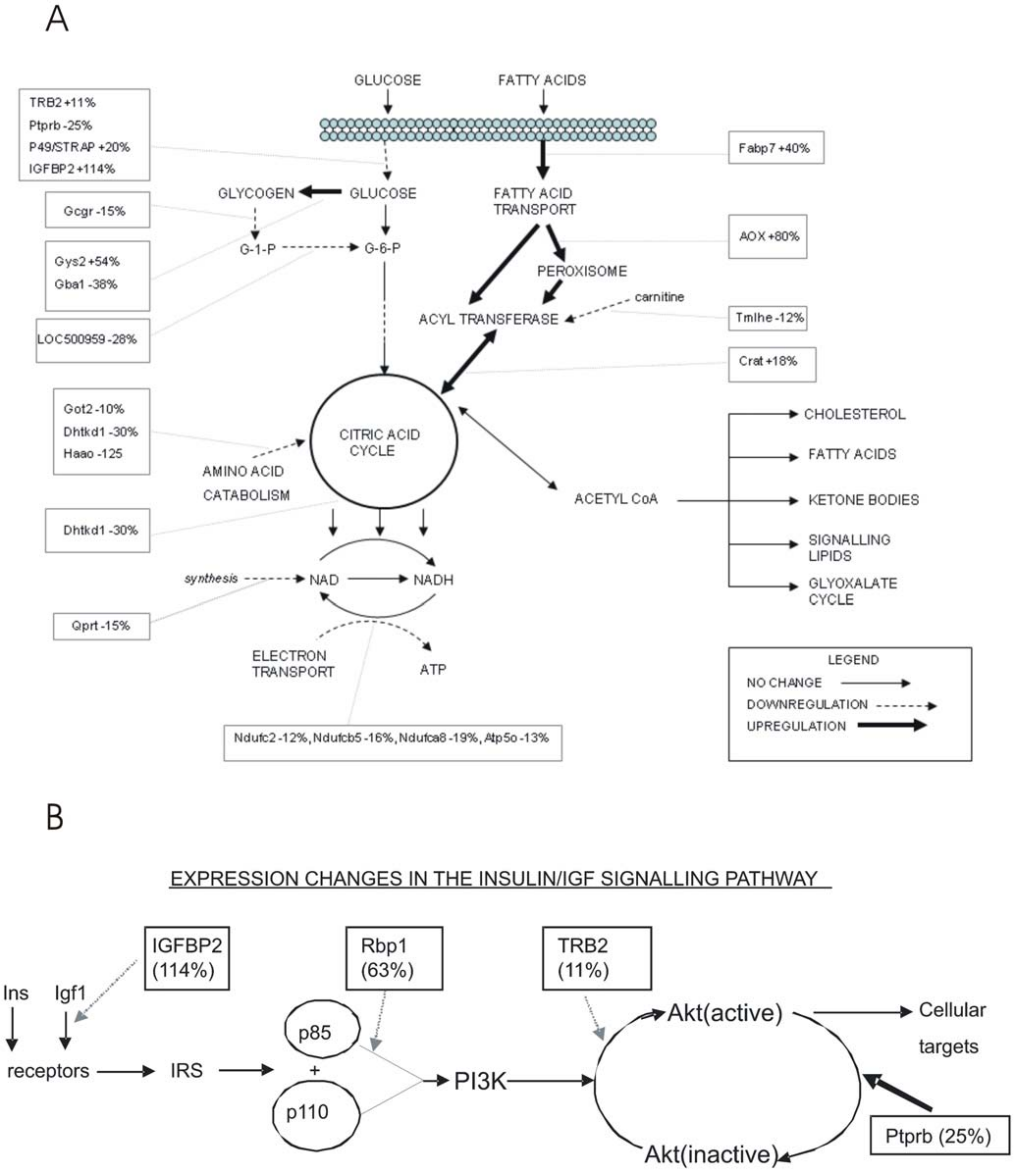
Genes that Influence Glucose, Fat, and Energy Metabolism Pathways and IGF-1 and PI3K Signaling Pathways. (A) Summarizes the interrelationships between pathways involved in glucose, fat, and energy metabolism and highlights differentially regulated genes that influence these pathways. (B) A summary of the genes that are altered in signaling through the IGF-1 and PI3K signaling pathways and their relevance to glucose and fat metabolism. Normal arrows indicate no change in gene expression; dashed arrows indicate down regulation; bold arrows indicate up-regulation.

Analysis of the gene categories and associated pathways associated with differentially expressed genes (Table S3) has revealed some relevant changes that potentially impact on the metabolic phenotype. Table 4 summarizes the relevant categories to emerge from analysis of gene functions (some of which emerged from DAVID and Ingenuity analyses) associated with the list of differentially expressed genes. Figure 2A and 2B summarize the interrelationships between pathways involved in glucose, fat, and energy metabolism and highlight differentially regulated genes that influence these pathways.

**Table 4:** Analysis of gene function associated with differentially expressed genes.

METABOLIC AREA	SYMBOL	GENE IDENTIFICATION	% ΔEXP	FUNCTION
Carbohydrate and Glucose metabolism	Gys2	glycogen synthase	54	stimulation of glucose storage
	Gbe1	glucan 1,4α branching enzyme	−38	impairment of glycogen storage
	Pgm1	phosphoglucomutase	−14	increases glycogenesis over glycolysis
	Tpi	triose phosphate isomerase	−28	attenuation of glycolysis and energy production
	Gcgr	glucagon receptor	−15	attenuation of glycogenolysis
	Trib3	Tribbles homolog 3	11	impairment of insulin/IGF signaling at Akt [46]
	Ptprb	protein tyrosine phosphatase receptor type B	25	impairment of insulin/IGF signaling at Atk [47]
	Rbp1	Retinol binding protein 1	63	impairment of insulin/IGF signalling at PI3Kinase [30]
	Igfbp2	insulin-like growth factor binding protein 2	114	impaired IGD binding to receptor
	p49/STRAP	SRF-dependent transcription regulation associated protein	10	interference with GLUT4 biosynthesis and recycling [48]
	TRIP3	thyroid hormone interactor protein 3 (TRIP3)	6	activator of NHF-4α dependent effects on glucose metabolism [49]
	Dhtkd1	dehydrogenase E1 and transketolase	30	catalyses αketoglutarate→succinylCoA in TCA
Fat and Lipid Metabolism	Crat	carnitine acyltransferase	18	increased transport of acylCoA from cytosol to mitochondrion
	Tmhle	trimethyllysine hydroxylase	−12	biosynthesis of carnitine
	Dgat2	diacylglycerol acyltransferase	−12	decreased synthesis of triglycerides
	Fabp7	fatty acid binding protein 7	40	increased uptake and intracellular transport of fatty acids
	Scap	SREBP cleavage activating protein (Scap)	20	increased hepatic lipid synthesis [50]
	Igfbp2	IGF binding protein 2	114	protection against obesity and insulin resistance [51]
	Ptprb	protein tyrosine phosphatase receptor type B	25	contributes to hepatic leptin resistance [52]
	Stard5	steroidogenic acute regulatory protein	−12	intracellular cholesterol transport
	Cyp4f4	cytochrome P450	−28	involved in cholesterol, steroid and leukotriene synthesis
	Cyp4f6	cytochrome P450	−18	involved in cholesterol, steroid and leukotriene synthesis
	Atp8b1	ATPase class II type 8B	16.5	phosphatidyl serine and threonine transferase
	Cidea	cell death inducing DNA fragmentation factor α-like effector	−28	shown to regulate energy expenditure and lipolysis [53], [54], [55], [56]
	Ebp	phenyalkylamine Ca2+ antagonist (emopamil) binding protein	20	isomerase involved in the conversion of lanosterol to cholesterol
Amino Acid Metabolism	Got2	glutamate/oxaloacetate transaminase	−10	aspartate aminotransferase in aa catabolism and malate shuttle
	Dhtkd1	dehydrogenaseE1 and transketolase domain containing 1	−30	2-ketoglutarate dehydrogenase activity
	Hpd	4-hydroxyphenylpyruvate oxidase	16	participates in tyrosine catabolism
	Haao	3-hydroxyanthanillate dioxygenase	−12	participates in tryptophan catabolism
Protein turnover	Zfand2a	arsenite inducible RNA associated protein	40	adapts proteosome to counteract stress-induced proteotoxicity [57]
	Tmprss6	transmembrane serine protease 6	−15	
	Capn7	calpain 7	−15	
	Cpn2	carboxypeptidase N83	−58	
	Capsn1	calpain small subunit 1	−21	
	Fam108b1	Cgi67 serine protease	−2.8	
	Serpinb6b	serine(or cysteine) peptidase inhibitor,clade B, member 6	25	
	Cand1	cullin associated/neddylation dissociated 1	15	involved in targeted protein proteosomal degradation [58]
	Dcun1d5	defective in cullin neddylation1, domain 5 (DCN1)	13	involved in targeted protein proteosomal degradation [59]
	Txndc12	Thioredoxin domain containing 12 (Txndc12)	20.6	protein refolding, defense against oxidative stress
	Ubfd1	ubiquitin family domain containing 1 (Ubfd1)	14	involved in targeted protein proteosomal degradation
	Eif4a1	translation initiation factor 4A1	20	
Ribosomes	Rpl7a	ribosomal protein L7a (mitochondrial)	24	
	Rps19	ribosomal protein S19	22	
	Rpl14	ribosomal protein L14 (mitochondrial)	20	
	Rps26	ribosomal protein S26 (mitochondrial)	19	
	Rp23a	ribosomal protein 23a (mitochondrial)	19	
	Rpl27a	ribosomal protein 27a (mitochondrial)	18	
	Rpl21	ribosomal proetin L21(mitochondrial)	17	
	Rpl31	ribosomal protein L31	17	
	Rpl39	ribosomal protein L39	15	
	Rpl6	ribosomal protein L6	14	
	Rps17	ribosomal protein S17 (mitochondrial)	14	
	Rpl24	ribosomal protetin L24	14	
	Rpl13	ribosomal protein L13 (mitochondrial)	7	
	NPG1	autoantigen NPG1	16	required for the maturation/nuclear export of pre-ribosomes
	Bxdc1	brix domain containing 1	11	control of ribosome biogenesis [60]
	Crlz1	charged amino acid leucine zipper 1	26	required for 18s rRNA processing
	Rrp15	ribosomal protein processing homolog 15 (Rrp15)	17	
	Cebpz	CCAAT/enhancer binding protein zeta	17.4	Ribosomal RNA processing [35]
Apoptosis	Dnase1l3	DNAse 1-like 3	40	fragments DNA during apoptosis
	Ppid	peptidyl prolyl isomerase D (Cyclophilin D)	20	anti-apoptotic. Blocks mitochondrial permeability
	Ei24	etoposide induced 2.4 mRNA	17	may suppress cell growth by inducing apoptosis
	Prkch	protein kinase C eta	14	anti-apoptotic. Potent activator of Raf1
	Sgpl1	sphingosine phosphate lyase	18	apoptotic. Enhances stress-induced ceramide release [61]
	Aatf	apoptosis antagonising factor	13	anti-apoptotic nuclear phosphoprotein transcription factor
	Cidea	cell death inducing DNA fragmentation factor α-like effector	−28	apoptotic factor which induces DNA fragmentation [53], [54], [55], [56]
	SP16	serpin 9b protease inhibitor	27	regulates apoptosis by inhibiting caspases 8 and 10
Mitochondrion and Electron Transport Chain	LOC499612	NADH dehydrogenase (ubiquinone) complex unknown	−11	uncharacterised component of electron transport chain
	Ndufc2	NADH dehydrogenase (ubiquinone) 1 complex unknown 2	−12	uncharacterised component of electron transport chain
	Ndufc6	NADH dehydrogenase (ubiquinone) 1 beta subcomplex 6	−16	component of Complex I of electron transport chain
	Ndufa8	NADH dehydrogenase (ubiquinone) 1 alpha subcomplex 8	−19	component of Complex I of electron transport chain
	p45/STRAP	SRF-dependent transcription regulated associated factor	−10	reported to interfere with Comlpex I assembly
	BC088177 - Qprt	quinolate phosphoribosyl transferase	−29	participates in NAD biosynthesis
	Qprt	quinolate phosphoribosyl transferase	−15	participates in NAD biosynthesis
	Atp5o	ATP synthase H ion transporting	−14	part of the mitochondrial ATP synthesis complex
	Atpi	ATPase inhibitor	−12	prevents ATP hydrolysis during electron flux
	Ppid	peptidyl prolyl isomerase D (Cyclophilin)	−20	part of mitochondrial transition pore
	Tim14	translocase complex subunit Tim14	−29	component of the mitochondrial protein import motor
	Haao	3-hydroxyanthanillate dioxygenase	−12	provides quinolate for NAD synthesis
Transcription	Crem	cAMP responsive element modulator	27	PKA activation. Role in glucose and fat metabolism
	Gtf2a2	general transcription factor IIa 2	12.5	part of the preinitiation transcription complex
	Gtf2hI	general transcription factor II H, polypeptide 1	3	part of the preinitiation transcription complex
	MED21	mediator complex subunit 21	11	part of the RNA polymerase II transcription unit
	MED6	mediator complex subunit 6	9.3	part of the RNA polymerase II transcription unit
	Rbp1	retinoic acid binding protein	63	RXR formation and PPAR activation
	DDX52	DEAD box polypeptide 52	18.7	ATP-dependent RNA helicase
	Ccdc12	Ccdc coiled-coil domain containing 12	14	involved in mRNA splicing

Summarizes the interesting categories to emerge from analysis of gene functions (some of which emerged from DAVID and Ingenuity analyses) associated with the list of differentially expressed genes.

### Glucose Metabolism

*In utero* the fetus relies mainly on maternal glucose for its energy supply and only in late gestation develops the capacity for glucose storage and mobilization and for gluconeogenesis in preparation for postnatal life. Glucose and glycogen metabolism appear to have developed inappropriately in the livers of UN animals [26], [27]. Many of the changes in hepatic gene expression we detected are relatively modest and this may reflect alterations in hepatic development. Burns *et al.* reported changes in the balance of periportal to periarterial hepatocytes which have different metabolic profiles in the offspring of low protein fed dams [28].

The livers of UN animals show changes in the anomalous expression of RNA for several enzymes involved in glycogenesis, glycogenolysis and glycolysis. There is a 54% increase in the expression of the glycogen synthase gene (GYS2), however, this is counterbalanced by a marked decrease in expression of glucan branching enzyme (GBE1) that is essential for the formation and storage of glycogen. Furthermore, there is a modest decrease in phosphoglucomutase (PGM1) that converts the glucose-1-phosphate resulting from glycogenolysis to glucose-6-phosphate destined for glycolysis. There is also a deficit of triose phosphate isomerase that could result in an inefficient conversion of fructose 1–6 bisphosphate to glyceraldehyde-3 phosphate in the glycolytic process. The reduced expression of triose phosphate isomerase would therefore affect the efficiency of glucose conversion to pyruvate and might indicate an increased accumulation of dihydroxyacetone phosphate. This can readily be reduced to glycerol-3-P that is the backbone for phosphatidates and signaling phospholipids. Overproduction of these could disrupt intracellular function. The efficiency of the citric acid cycle (TCA) would be compromised by the reduction in dehydrogenase E1 (DHTKD1). This enzyme catalyses the conversion of α-ketoglutarate to succinyl CoA- a key step in the TCA. Its subsequent conversion to succinate is the only step in glucose catabolism that produces high-energy phosphate (GTP) directly, without the need for oxidative phosphorylation. The GTP can be used for nucleotide-specific metabolic reactions or it can convert ADP to ATP. Production of oxaloacetate further on in the cycle would also be affected. This would slow the rate of formation of citric acid and the entry into the TCA of acetyl-CoA from oxidative decarboxylation of pyruvate. Supplementation of oxaloacetate from metabolism of branched-chain amino acids would also be reduced. This means that most of the oxaloacetate is required as an intermediate for TCA function and less is available for gluconeogenesis. It is interesting to note that there is a reduction in the transcripts of other enzymes involved in the catabolism of amino acids that supply intermediates to the TCA cycle. In the fetus, this impasse may be circumvented to an extent by the presence of the glyoxalate cycle in which acetate can be converted to either glucose or succinate. However, this pathway disappears after birth and hence the inability for gluconeogenesis would be exacerbated postnatally.

### Insulin and IGF-1 Signalling

The insulin and IGF-1 receptors are closely related structurally and share many post-receptor signaling mechanisms. The livers of UN animals show changes in expression of genes whose products are known to attenuate the PI3 kinase signaling pathway that is common to both receptors. Both IGFBP2 and Rbp1 are known to interact with components of the PI3Kinase/Akt signaling pathway IGFBP2 is likely to act by sequestering IGF-1 and preventing binding to its receptor and hence inhibiting downstream activation of PI3K [29]. Rbp1 has been shown to interfere with the heterodimerization of the p85/p110 subunits of Akt [30]. This appears to be a retinoic acid independent effect of Rbp1. An increase in expression of Tribbles homolog 2 (TRB2) has been shown to interfere with the phosphorylation step by PI3K that activates Akt, the effector kinase of this signaling cascade. Conversely increased expression of the protein tyrosine phosphatase (PTPRB) will increase the rate of dephosphorylation of Akt to its inactive form. The concerted effect of these gene expression changes is likely to have the effect of blunting insulin signaling and increasing insulin insensitivity. It is interesting to note that while there is not a significant increase in the levels of circulating insulin, there is a discernable upward trend in UN animals that could indicate the initial stages in the establishment of insulin resistance. One of the major targets of Akt is the insulin-dependent glucose transporter GLUT4 that on activation migrates from the ER to the plasma membrane and facilitates glucose entry into the cell. An additional perturbation of this process in UN animals is suggested by the increased expression of p45/STRAP that has been shown to interfere with GLUT4 [26], [31] trafficking between the ER and the cell membrane. Taken together these findings suggest that the livers of UN animals have altered insulin sensitivity and glucose homeostasis.

A primary function of IGF-1 is to drive postnatal cell proliferation and growth, principally through the MAP kinase signaling pathway. It is only present in low levels in the fetus (where IGF-2 predominates) and increases dramatically in the perinatal period. The liver is the major source of circulating IGF-1. The actions of IGFs are modulated by the presence of a number of binding proteins (IGFBPs), one of which IGFBP2, shows a >2 fold increase in expression in liver of UN animals. IGFBP2 is secreted into the blood and is thought to have a negative effect on IGF-1 activity. In this study, the highly significant reductions in body length and body, spleen, and liver weights lend support to this role. The levels of IGFBP2 have been shown to be high in the early to mid-term fetus and then drop markedly towards term. The persistence of elevated IGFBP2 in UN animals is therefore an abnormality that could have a wide range of effects on postnatal growth and organ development.

### Mitochondrial Activity

The array analysis indicates a number of changes in the expression of genes that could affect the efficiency of electron transport and ATP generation in the mitochondrion in liver from UN animals. Transcripts of NADH dehydrogenase subunits (NDUFC2, NDUFB6 and NDUFA8) of complex I of the electron transport chain are all reduced as is quinolate phosphoribosyltransferase (QPRT) required for the synthesis of NAD, the electron carrier. In addition, the ATP synthase (ATP5o) that generates ATP from the electron gradient is reduced by a similar amount. Changes are also observed in peptidylprolyl isomerase D (PPID) that controls the mitochondrial permeability transition pore and TIM14, a component of the mitochondrial protein import motor. Taken together these findings suggest a degree of mitochondrial dysfunction in the livers of UN animals that could significantly affect the energy balance of the tissue and may be the precursor of later adiposity. Since the liver acts as the initial nutrient sensor, this defect could affect not only its own metabolism but also that of other organs that respond to its systemic signals.

### Fat

In this well-characterized model, it has been shown that undernourishment of dams during pregnancy predisposes offspring to later adiposity and hyperinsulinaemia when fed a normal diet [21] but only at an age later than we have undertaken gene expression analysis. In the present study, even as early as day 110, male offspring of UN dams are showing features of the metabolic syndrome related to increased adiposity and hyperinsulinemia and hyperleptinemia. The absence of any differences in fat expression profiles between control and UN animals at day 55 may suggest that the development of adiposity is secondary to early metabolic perturbations in postnatal life. It is interesting to note that at day 35 UN rats show the onset of a sedentary phenotype (and hence reduced energy expenditure) before the presence of obesity [32]. This may reflect early changes in neurological gene expression modifying behaviour.

The elevated expression of RNA for fatty acid binding protein (FABP7) suggests that in livers from UN animals there is an increase in the intracellular trafficking of fatty acids. This is paralleled by increases in expression of AOX (from qRT-PCR analysis), which catalyses the first step in the peroxisomal degradation of fatty acids, and carnitine acyltransferase (CRAT) that shuttles fatty acids into the mitochondrial matrix. It is interesting to note that the expression of the first enzyme in the biosynthetic pathway of carnitine, trimethyllysine hydroxylase (Tmlhe) is down regulated and this may reflect a homeostatic response to excess fatty acid trafficking. These findings suggest that more energy is derived from β-oxidation of fatty acids in these animals. However, this can only be achieved if the NADH from β-oxidation regenerates NAD and forms ATP. The observed defects (especially in complex I) indicate a disruption in this process. The provenance of these fatty acids is not clear since there were no significant differences between treatment groups in the circulating levels of either triglycerides or free fatty acids although LDL levels are elevated by maternal undernutrition. Other possible sources of fatty acids could be from increased endogenous synthesis or from turnover of cellular components. There is no evidence from the microarray analysis for an increase in enzymes for the latter; indeed the modest decrease in levels of diacylglycerol acyltransferase (DFAT2) in UN animals would indicate a blunting of triglyceride synthesis in liver.

The increased expression of SREBP cleavage activating protein (SCAP) suggests that there may be higher levels of sterol synthesis taking place in livers of undernourished animals. The formation of a transcriptionally active sterol response element binding protein (SREPB) involves the proteolytic action of SCAP [33]. In support of these observations the levels of circulating LDL cholesterol in the UN group is significantly elevated. Interestingly a 60% increase in 11β-HSD2 transcripts was seen in the livers of UN animals accompanied by a similar increase in the expression levels of GR. 11β-HSD2 appears in late gestation/postnatal tissues in human fetal tissues. [34] Elevated levels of this enzyme in the livers of UN animals would be a mechanism to moderate the effects of high levels of circulating corticosterone that may arise in response to nutritional deprivation/stress.

### Ribosomal Proteins and Protein Turnover

An intriguing finding of the analysis is an increase (in the range 7–24%) in transcripts in UN animals of 13 of 80 recognized ribosomal proteins (79 of which are represented on the array by multiple oligonucleotides) that occur in the large and small ribosomal subunits. Eight of the proteins are components of the mitochondrial ribosome. Similar increases in expression are also seen in brix (BXDC1), involved in ribosome biogenesis, NPG1, a nucleolar GTPase required for the maturation and nuclear export of pre-ribosomes, and Cebpz, a gene important in the processing of rDNA involved in the 60 s subunit [35]. These findings suggest a greater degree of metabolic flux in ribosomes from UN animals, however it is difficult to understand the significance of these observations given the fact that on the basis of the current annotation only about 16% of the ribosomal proteins show changes in expression levels. It is possible that the increased turnover of ribosomal proteins is contributing to the higher levels of urea observed in the day 55 UN animals.

### Effects on Gene Transcription

It is notable that a group of genes that influence transcription show upregulation to varying extents. Some (such as the Gtf and MED factors) have a general effect on the transcriptional apparatus. The most notable increase is in the Rpb1 gene known to be expressed highly in the liver, particularly in hepatic stellate cells [36], [37]. In liver Rpb1 has an important role in the production of retinoid derivatives that activate the RAR and RXR retinoic acid receptors [38]. RXR can heterodimerise with the PPAR proteins to form active nuclear receptors that target genes involved in fat metabolism and energy homeostasis; for example Crat and AOX are shown to be upregulated in the UN offspring. Thus elevated or abnormal Rbp1 expression may have a major role in establishing the metabolic syndrome phenotype.

Two other genes show significant changes in gene expression that may impact hepatic function. The first is Col14a1 (a member of the collagen gene family) that was originally called undulin [39], [40]. This gene is expressed in hepatic stellate cells that have a role in the formation of the hepatic extra cellular matrix [41] and shows a 20% increase in gene expression. This may be a marker of some underlying pathology as Col14a1 has been associated with the rearrangement of connective tissue occurring in hepatic fibrosis [39]. It is of note that Rbp1 expression changes have also been associated with hepatic fibrosis [37] and thus the gene may be involved in the regulation of Col14a1. The second gene is Adra1b (adrenergic receptor alpha 1b showing a 79% upregulation) that has been associated with carbohydrate metabolism in the liver [42] and disruption of glucose homeostasis when the gene is inactivated [43], [44]. Given that the role of the receptor is to stimulate glycogenolysis and the process of glycogen formation is impaired in UN animals, the significance of increased expression is not clear. This may reflect an irreversible change in response to the intrauterine nutritional state or a postnatal compensatory mechanism to the altered metabolic profile set *in utero*.

### Conclusions

Studies to date have primarily focused on a candidate gene approach in animals where the classical features of the metabolic syndrome in the programmed phenotype are already evident. There is a relative paucity of data on gene expression, either by candidate or an array-based approach, on key tissues related to the metabolic syndrome in animals at an age preceding development of the metabolic phenotype. The gene expression changes in appropriate pathways in the livers of the male offspring of maternally undernourished dams at day 55 would suggest that these animals may be predisposed to a persistent and perturbed ability to coordinate fat and carbohydrate metabolism with a shift to a use of fatty acids as an energy source. We have also shown that these animals proceed to develop a phenotype similar to that of the metabolic syndrome as early as postnatal day 110. Array analysis of tissues at the day 110 time point was beyond the scope of the present trial. However, although the present study cannot directly correlate the observed gene expression changes at day 55 with the phenotype at day 110, it provides clear evidence of disturbed hepatic function in a number of key genes related to lipid oxidation and mitochondrial function at a pre-phenotypic age. There are parallels with the observations of Koves et al. [27] and Sparks et al. [45] where diet induced obesity in adult life induces oxidative stress associated with increased β-oxidation of fat metabolism, impaired switching of carbohydrate substrates, depletion of TCA cycle intermediates, and decreased expression of components of the electron transport chain. They propose that mitochondrial dysfunction may be driving the pre-diabetic insulin resistant state. The observations in our rat model indicate that the liver is irreversibly programmed to respond to a nutritionally restricted environment and that this persists into early adulthood (day 55). At this age, similar changes are not observed in adipose tissue or skeletal muscle, suggesting that the liver can meet the immediate energy requirements of these peripheral tissues and that it manifests metabolic abnormalities in advance of the full metabolic syndrome phenotype. These data have uncovered potential candidate genes and pathways that when perturbed lead to the development of the metabolic syndrome in older animals and therefore provide a focus for more detailed gene-specific studies.

## Supporting Information

Table S1 PCR primer sequences used for the qRT-PCR analysis
(0.03 MB XLS)

Table S2 Raw data for all Illumina Array Data after filtering and normaization including annotation
(7.06 MB XLS)

Table S3 Log Fold Change, Adjusted p-value, Accession Number, and Gene Symbol for the 249 differentially expressed genes (p-value<0.05).
(0.07 MB XLS)

Figure S1 Illumina internal quality control measurements with BeadStudio Software for all 48 arrays. (A) Arrays should have signal intensities in the same range. There are three categories of signal intensity: low, medium, and high. A linear increase from low to medium to high is expected. (B) This image shows the individual medium intensity values for each array. Arrays should have signal intensities in the same range. (C) Compares the signal intensity of two mismatches to the signal intensity of a perfect match. The perfect matches should have a 3–4 fold higher signal. No signal intensite would indicate a hybridization failed. (D) Shows the high stringency of the data The biotin signal should be 3–4 fold lower than the high stringency signal. (E) This image shows the data for the negative control. The background signal should be approximately 70. The noise signal should be very low for high quality data. (F) This image compares the signal from the housekeeping genes to the signal from all genes. As housekeeping genes are always expressed they should have a much higher signal compared to all genes.
(1.28 MB TIF)

Figure S2 Visualization of Raw and Normalized Microarray Data. Graphs are shown of raw data (A, B) and normalized (C, D) data for each of the 64 microarrays. (A)Boxplot of the amplitude for the array signal for the raw data. (B) Density plot of the intensity of the raw data for each array. (C) Boxplot of the amplitude for the array signal for the normalized data. (C) Density plot of the intensity of the normalized data for each array.
(2.22 MB TIF)
