# Supplementary figures and images for: Transcriptional Profiling of Rats Subjected to Gestational Undernourishment: Implications for the Developmental Variations in Metabolic Traits
Source: PLoS One. 2009 Sep 29;4(9):e7271. doi: 10.1371/journal.pone.0007271 (PMC2749934; doi:10.1371/journal.pone.0007271)

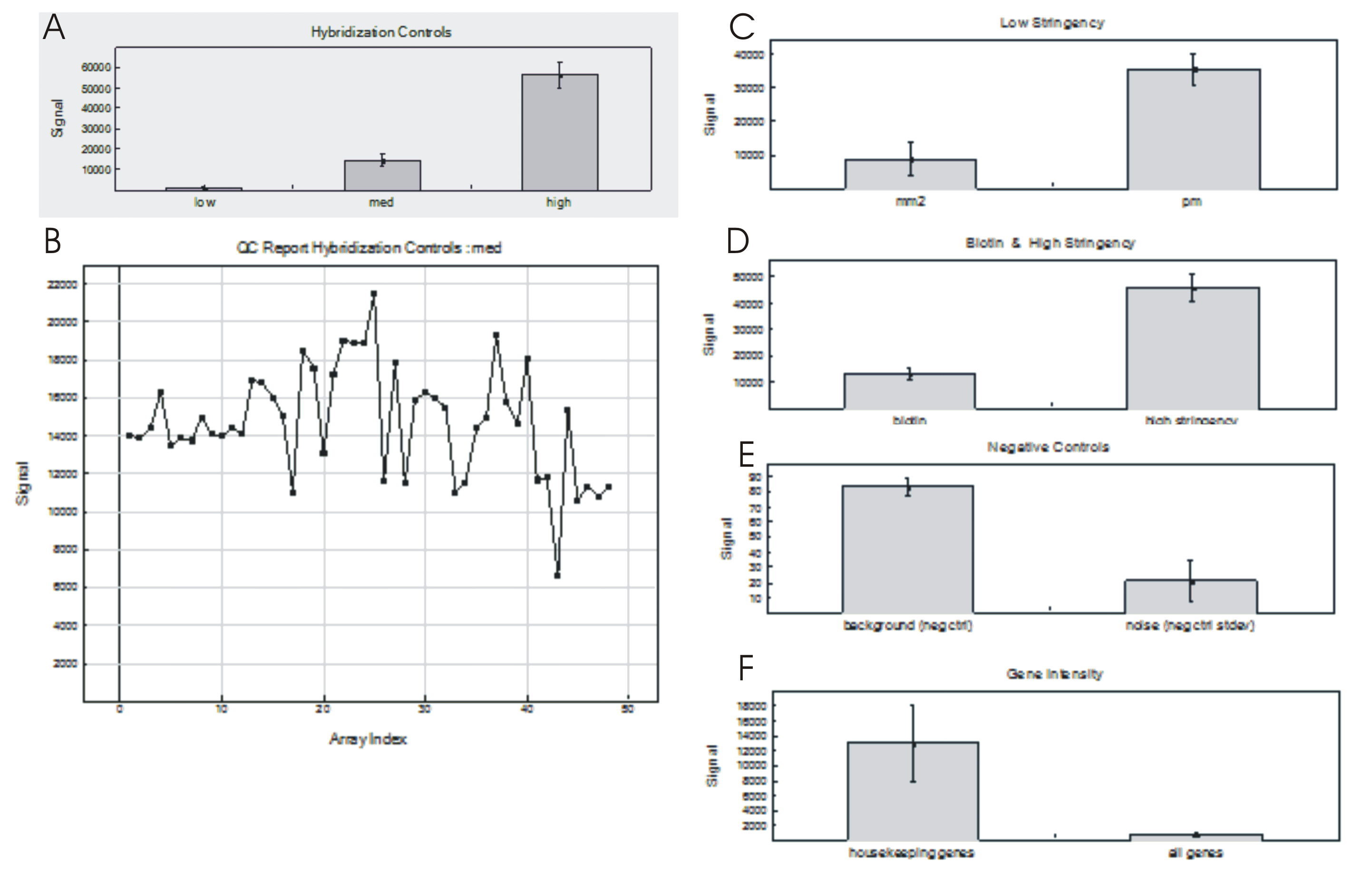

Supplement: Figure S1 — Illumina internal quality control measurements with BeadStudio Software for all 48 arrays. (A) Arrays should have signal intensities in the same range. There are three categories of signal intensity: low, medium, and high. A linear increase from low to medium to high is expected. (B) This image shows the individual medium intensity values for each array. Arrays should have signal intensities in the same range. (C) Compares the signal intensity of two mismatches to the signal intensity of a perfect match. The perfect matches should have a 3–4 fold higher signal. No signal intensite would indicate a hybridization failed. (D) Shows the high stringency of the data The biotin signal should be 3–4 fold lower than the high stringency signal. (E) This image shows the data for the negative control. The background signal should be approximately 70. The noise signal should be very low for high quality data. (F) This image compares the signal from the housekeeping genes to the signal from all genes. As housekeeping genes are always expressed they should have a much higher signal compared to all genes. (1.28 MB TIF) [file pone.0007271.s004.tif]

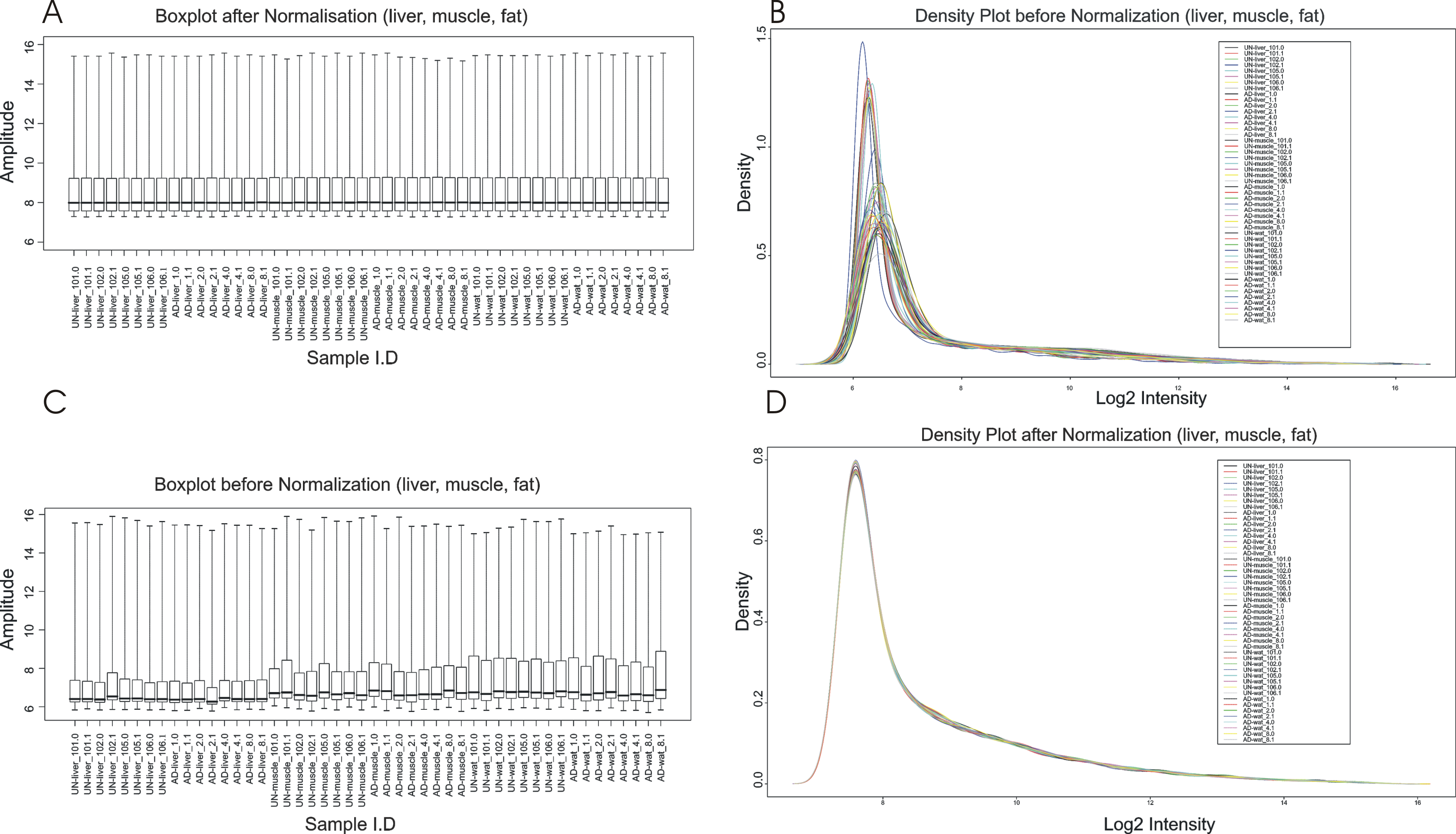

Supplement: Figure S2 — Visualization of Raw and Normalized Microarray Data. Graphs are shown of raw data (A, B) and normalized (C, D) data for each of the 64 microarrays. (A)Boxplot of the amplitude for the array signal for the raw data. (B) Density plot of the intensity of the raw data for each array. (C) Boxplot of the amplitude for the array signal for the normalized data. (C) Density plot of the intensity of the normalized data for each array. (2.22 MB TIF) [file pone.0007271.s005.tif]
